# Comparative Efficacy and Safety of Tirzepatide Versus Semaglutide for Obesity: A Systematic Review

**DOI:** 10.7759/cureus.114454

**Published:** 2026-08-13

**Authors:** Samih Abdelmutalab Mohamed Abdalla, Lima Gharbawi, Namaa Elshaikh, Eltayeb Osman Elfaki Omer, Alaa Fathelrahman Mokhtar Osman, Asjed Salaheldin Hassan Ali, Tasneem Mohamed Yousif Mohamed, Ali Hadi M Alhajri

**Affiliations:** 1 Internal Medicine, Al Hammadi Hospital, Riyadh, SAU; 2 ENT, Portsmouth University Hospitals NHS Trust, Hampshire, GBR; 3 Internal Medicine, University of Khartoum, Khartoum, SDN; 4 Endocrinology, Dr. Sulaiman Al Habib Hospital, Riyadh, SAU; 5 Internal Medicine, University Hospital Kerry, Kerry, IRL; 6 General and Colorectal Surgery, South Tipperary General Hospital, Clonmel, IRL; 7 Endocrinology, Najran Armed Forces Hospital, Ministry of Defense Health Services, Najran, SAU

**Keywords:** comparative effectiveness, gip, glp-1 receptor agonist, obesity, semaglutide, systematic review, tirzepatide, weight loss

## Abstract

Tirzepatide, a dual glucose-dependent insulinotropic polypeptide (GIP) and glucagon-like peptide-1 (GLP-1) receptor agonist, and semaglutide, a selective GLP-1 receptor agonist, are the two most effective approved injectable pharmacotherapies for obesity. Until recently, comparisons between them rested largely on cross-trial inference. A direct head-to-head randomised trial and a rapidly expanding body of comparative real-world evidence now permit a formal appraisal.

This objectives of this review are to systematically identify, appraise, and synthesise original comparative studies evaluating the efficacy and safety of tirzepatide versus semaglutide in adults with overweight or obesity.

This review followed the Preferred Reporting Items for Systematic Reviews and Meta-Analyses (PRISMA) 2020 statement. PubMed/MEDLINE, Embase, Scopus, and Web of Science were searched up to 20 July 2026, with backward and forward citation tracking. Eligible studies were peer-reviewed original randomised or non-randomised comparative studies directly comparing subcutaneous tirzepatide with subcutaneous semaglutide in adults with overweight or obesity and reporting at least one anthropometric outcome; non-original publications were excluded. Risk of bias was assessed with RoB 2 and Risk Of Bias In Non-randomised Studies - of Interventions (ROBINS-I). Substantial heterogeneity precluded meta-analysis, and findings were synthesised narratively.

Of 535 records identified, 11 studies comprising approximately 62,700 analysed participants were included: one phase 3b open-label randomised active-controlled trial and 10 retrospective observational cohorts from the United States, Kuwait, Turkiye, Bangladesh, and an international federated network. Tirzepatide produced greater weight reduction than semaglutide in every study that formally tested the comparison. In the randomised trial, mean weight change at 72 weeks was -20.2% with tirzepatide versus -13.7% with semaglutide. In real-world cohorts, adjusted between-group differences ranged from approximately 2.3 to 4.4 percentage points, and the advantage was most pronounced at stringent weight-reduction thresholds. Tirzepatide was also associated with greater improvements in blood pressure and glycated haemoglobin and a lower incidence of new-onset type 2 diabetes. Gastrointestinal events predominated with both agents. The randomised trial and six cohorts were judged at low risk of bias, and four cohorts at serious risk, principally from unadjusted confounding.

Tirzepatide achieves greater weight reduction than semaglutide in adults with overweight or obesity, with a broadly comparable short-term safety profile. The advantage is attenuated in routine care relative to the trial setting, and long-term head-to-head data on cardiovascular and other clinical endpoints remain absent.

## Introduction and background

Obesity is a chronic, progressive, and relapsing disease that has become one of the defining public health challenges of the current era. A pooled analysis of 3,663 population-representative studies encompassing 222 million children, adolescents, and adults across 200 countries and territories documented that the age-standardised prevalence of obesity more than doubled in adult women and nearly tripled in adult men between 1990 and 2022, and that obesity has now overtaken underweight as the dominant form of malnutrition in most countries [1]. Excess adiposity is causally implicated in type 2 diabetes, hypertension, dyslipidaemia, obstructive sleep apnoea, metabolic dysfunction-associated steatotic liver disease, osteoarthritis, and several cancers, and it substantially impairs health-related quality of life and life expectancy. The economic consequences are correspondingly large: a cost-of-illness analysis spanning 161 countries estimated that the direct healthcare and indirect productivity costs of overweight and obesity amounted to 2.19% of global gross domestic product in 2019 and projected a rise to 3.29% by 2060 under current trends, with the steepest proportional increases expected in low- and middle-income countries [2]. Effective and durable treatment is therefore a public health as well as a clinical priority. Although structured lifestyle modification remains the foundation of obesity care, it seldom produces the magnitude or durability of weight reduction required to reverse obesity-related complications, and contemporary clinical guidance therefore positions pharmacotherapy as an integral component of comprehensive management for adults with a body mass index (BMI) of at least 30 kg/m², or at least 27 kg/m² in the presence of a weight-related complication [3].

The therapeutic landscape has been transformed by incretin-based pharmacotherapy. Incretins are gut-derived hormones released after eating that stimulate insulin secretion, suppress appetite, and slow gastric emptying; the two principal incretins are glucagon-like peptide-1 (GLP-1) and glucose-dependent insulinotropic polypeptide (GIP). Semaglutide is a long-acting analogue that activates the GLP-1 receptor alone, whereas tirzepatide is a single molecule that activates both the GIP and GLP-1 receptors, and this dual mechanism provides the pharmacological rationale for expecting greater weight loss. In trials of adults with overweight or obesity but without diabetes, once-weekly subcutaneous semaglutide 2.4 mg reduced mean body weight by 14.9% at 68 weeks in STEP 1 [4], while tirzepatide reduced it by 15.0% to 20.9% across its 5 mg, 10 mg, and 15 mg doses at 72 weeks in SURMOUNT-1 [5]. In the parallel trials enrolling adults with coexisting type 2 diabetes, in whom weight loss is characteristically attenuated, semaglutide 2.4 mg achieved a 9.6% reduction in STEP 2 [6] and tirzepatide achieved 12.8% to 14.7% in SURMOUNT-2 [7]. These figures come from separate trials with different populations and protocols and therefore cannot be compared directly, but they illustrate that the relative performance of the two agents may differ according to diabetes status. Beyond weight, semaglutide 2.4 mg reduced major adverse cardiovascular events by 20% in adults with overweight or obesity and established cardiovascular disease without diabetes in the SELECT trial [8], and the randomised withdrawal design of SURMOUNT-4 demonstrated that continued tirzepatide treatment is required to maintain weight reduction, since substantial regain follows discontinuation [9].

It is important to distinguish the earlier diabetes-focused comparison of these two agents from the obesity-specific question addressed here. For several years the only head-to-head randomised evidence was SURPASS-2, in which tirzepatide produced greater reductions in both glycated haemoglobin (HbA1c) and body weight than semaglutide [10]. That trial, however, enrolled adults selected on the basis of type 2 diabetes rather than excess adiposity, used glycaemic control as its primary endpoint, and compared tirzepatide against semaglutide 1 mg, the dose licensed for glycaemic management and well below the 2.4 mg dose licensed for weight management. SURPASS-2 therefore cannot be interpreted as a comparison of the two drugs at their obesity-approved doses, and the weight difference it reported may partly reflect the lower comparator dose rather than a difference in intrinsic efficacy. This limitation is precisely what made a dedicated appraisal of obesity-specific comparative evidence necessary.

Despite the scale of this evidence, a consequential uncertainty persisted: whether the apparently larger weight reductions seen in tirzepatide trials reflect genuine pharmacological superiority or merely differences in trial populations, background lifestyle intervention, comparator doses, and estimand definitions. Cross-trial comparison cannot resolve this question. That situation changed with the publication of SURMOUNT-5, the first randomised head-to-head comparison of the two agents at their maximum tolerated doses in adults with obesity but without type 2 diabetes, and with the near-simultaneous appearance of numerous comparative observational cohorts drawn from electronic health record and claims databases, specialist obesity clinics, and post-bariatric and diabetes populations across several continents. These studies differ markedly in population, follow-up duration, dose attainment, adherence requirements, and analytical rigour, and their findings have not previously been appraised together with formal risk-of-bias assessment in a way that distinguishes randomised from non-randomised evidence and trial-setting efficacy from routine-care effectiveness. The present systematic review was therefore undertaken to identify all eligible original comparative studies of tirzepatide versus semaglutide in adults with overweight or obesity, to summarise their efficacy and safety findings, to appraise their methodological quality, and to define the residual knowledge gaps that should shape the next generation of comparative research.

## Review

Materials and methods

Protocol and Reporting

This systematic review was designed, conducted, and reported in accordance with the Preferred Reporting Items for Systematic Reviews and Meta-Analyses (PRISMA) 2020 statement [11]. The review question was framed using the Population, Intervention, Comparator, Outcomes, and Study design (PICOS) structure, and the a priori eligibility criteria are presented in Table 1. In accordance with item 24 of the PRISMA 2020 checklist, we state explicitly that this review was not prospectively registered in PROSPERO, the Open Science Framework, or any other registry. The absence of a publicly accessible registration record is acknowledged as a limitation, since it prevents external verification that the reported methods and outcomes correspond to those planned.

**Table 1:** Eligibility criteria for the systematic review, structured according to the PICOS framework BMI: body mass index; GLP-1: glucagon-like peptide-1; HbA1c: glycated haemoglobin; PICOS: Population, Intervention, Comparator, Outcomes, Study design

PICOS domain	Inclusion criteria	Exclusion criteria
Population (P)	Adults aged 18 years or older with overweight (BMI 25.0-29.9 kg/m², or 27.0-29.9 kg/m² with a weight-related complication) or obesity (BMI at least 30 kg/m²). Cohorts with or without type 2 diabetes, with type 1 diabetes, and with weight recurrence after metabolic bariatric surgery were eligible provided the cohort was defined by excess adiposity.	Participants under 18 years of age; populations defined exclusively by a glycaemic indication; populations defined by an unrelated index condition in which excess adiposity was incidental; pregnant or lactating women; animal or in vitro studies.
Intervention (I)	Subcutaneous tirzepatide at any dose or formulation (Mounjaro or Zepbound), administered once weekly for weight management, on or off label.	Oral or investigational formulations; compounded or unregulated preparations; tirzepatide given as part of a fixed combination that could not be separately attributed.
Comparator (C)	Subcutaneous semaglutide at any dose or formulation (Ozempic or Wegovy) within the same study, permitting a direct within-study comparison. Additional comparator arms (for example liraglutide or usual care) were permitted if the two index arms were separately reported.	Comparison of either agent only against placebo, lifestyle intervention, bariatric surgery, or another drug; indirect comparison through a shared placebo; single-arm studies.
Outcomes (O)	Primary: absolute or percentage change in body weight, or proportion achieving categorical weight-reduction thresholds (at least 5%, 10%, 15%, 20%, or 25%). Secondary: BMI, waist circumference, blood pressure, HbA1c, lipids; incident type 2 diabetes; cardiovascular events; adverse events, dose attainment, and treatment persistence.	No anthropometric outcome reported for either arm; studies reporting only pharmacokinetic, biomarker, economic, prescribing-pattern, or qualitative outcomes.
Study design (S)	Peer-reviewed original randomised controlled trials or non-randomised comparative studies (prospective or retrospective cohort, case-control, or comparative cross-sectional) with a direct within-study comparison.	Ineligible designs; review articles, commentaries, and editorials; systematic reviews and meta-analyses; conference abstracts; case reports and series; protocols; preprints; post hoc analyses of an already included parent trial.
Timeframe and language	Published between 1 January 2021 and 20 July 2026. This window was prespecified in the protocol on pharmacological grounds: tirzepatide was first approved in 2022, so no direct comparative study could predate it. Reports in English.	Records published outside the search window; non-English reports; records for which the full text could not be retrieved.

Search Strategy and Information Sources

Four bibliographic databases were searched to 20 July 2026: PubMed/MEDLINE, Embase, Scopus, and Web of Science Core Collection. The search combined controlled vocabulary terms and free-text synonyms for the two interventions and for the population, using the following core string, adapted to the syntax of each database: (tirzepatide OR Mounjaro OR Zepbound OR "LY3298176" OR "dual GIP" OR "twincretin") AND (semaglutide OR Wegovy OR Ozempic OR "GLP-1 receptor agonist") AND (obesity OR obese OR overweight OR "weight loss" OR "weight reduction" OR "body weight" OR "body mass index" OR "weight management"). The full search string, date of execution, and filters applied in each database are reported in Table 2. A publication window of 1 January 2021 to 20 July 2026 was prespecified in the review protocol before the searches were executed. The rationale was pharmacological rather than empirical: tirzepatide first received regulatory approval in 2022, so no study directly comparing it with semaglutide could have been published before 2021, and the window therefore excludes no potentially eligible record. The window was not chosen on the basis of the number of studies retrieved. An English-language restriction was prespecified and applied as a filter in each database, a decision taken on the basis of the resources available for translation; the consequent potential for language bias is acknowledged in the Limitations. Grey literature was not searched, because unpublished and non-peer-reviewed sources, including conference abstracts and preprints, were excluded by the eligibility criteria; this decision and its implications are also addressed in the Limitations. The database searches were supplemented by backward citation tracking of all included studies and of relevant narrative and systematic reviews, and by forward citation tracking in Google Scholar and Web of Science. ClinicalTrials.gov was screened to identify completed but unpublished head-to-head studies; none were identified.

**Table 2:** Search strategy The same core string was applied in each database and adapted to its syntax. GIP: glucose-dependent insulinotropic polypeptide; GLP-1: glucagon-like peptide-1

Database	Search string	Date searched	Filters applied
PubMed/MEDLINE	(tirzepatide OR Mounjaro OR Zepbound OR "LY3298176" OR "dual GIP" OR "twincretin") AND (semaglutide OR Wegovy OR Ozempic OR "GLP-1 receptor agonist") AND (obesity OR obese OR overweight OR "weight loss" OR "weight reduction" OR "body weight" OR "body mass index" OR "weight management")	20 July 2026	Publication date 1 January 2021 to 20 July 2026; humans; English language
Embase	(tirzepatide OR Mounjaro OR Zepbound OR "LY3298176" OR "dual GIP" OR "twincretin") AND (semaglutide OR Wegovy OR Ozempic OR "GLP-1 receptor agonist") AND (obesity OR obese OR overweight OR "weight loss" OR "weight reduction" OR "body weight" OR "body mass index" OR "weight management")	20 July 2026	Publication date 1 January 2021 to 20 July 2026; humans; English language
Scopus	(tirzepatide OR Mounjaro OR Zepbound OR "LY3298176" OR "dual GIP" OR "twincretin") AND (semaglutide OR Wegovy OR Ozempic OR "GLP-1 receptor agonist") AND (obesity OR obese OR overweight OR "weight loss" OR "weight reduction" OR "body weight" OR "body mass index" OR "weight management")	20 July 2026	Publication date 1 January 2021 to 20 July 2026; document type: article; English language
Web of Science Core Collection	(tirzepatide OR Mounjaro OR Zepbound OR "LY3298176" OR "dual GIP" OR "twincretin") AND (semaglutide OR Wegovy OR Ozempic OR "GLP-1 receptor agonist") AND (obesity OR obese OR overweight OR "weight loss" OR "weight reduction" OR "body weight" OR "body mass index" OR "weight management")	20 July 2026	Publication date 1 January 2021 to 20 July 2026; document type: article; English language

Eligibility Criteria

Eligible records were peer-reviewed original research articles reporting a direct, within-study comparison of subcutaneous tirzepatide with subcutaneous semaglutide in adults aged 18 years or older with overweight or obesity, and reporting at least one anthropometric outcome (absolute or percentage change in body weight, change in BMI, achievement of categorical weight-reduction thresholds, or change in waist circumference). Both randomised controlled trials and non-randomised comparative studies were eligible, and studies including additional comparator arms were eligible provided that the tirzepatide and semaglutide arms were separately reported. Studies were excluded if the design was ineligible, if participants under 18 years of age were included, if the two agents were not compared directly for efficacy, or if the record was a review article, commentary, editorial, conference abstract, protocol, or preprint. Post hoc and secondary analyses of a parent trial already represented in the review were excluded to avoid double counting, with the primary report retained.

Study Selection

All records retrieved were imported into EndNote X9 (Clarivate Analytics, Philadelphia, PA), in which duplicates were removed electronically and then verified manually. Two reviewers independently screened titles and abstracts against the eligibility criteria, and the full texts of all potentially eligible records were then independently assessed. Disagreements were resolved by discussion and, where consensus was not reached, by adjudication from a third reviewer. Screening decisions were recorded in duplicate at both the title-and-abstract and full-text stages, and disagreements were infrequent and confined to the full-text stage; a formal chance-corrected measure of inter-rater agreement, such as Cohen's kappa, was not calculated prospectively, and this is acknowledged in the Limitations. Reasons for exclusion at the full-text stage were documented and are reported in the PRISMA flow diagram in the figure under the Results section.

Data Extraction

A standardised, piloted data extraction form was used. Two reviewers independently extracted the following from each included study: first author, publication year, country and setting, data source, study design, index period, sample size in each arm, participant age, sex distribution, baseline body weight and BMI, diabetes status, intervention and comparator with dose-attainment data, duration of follow-up, definition of the analysis population, statistical approach to confounding control, anthropometric and cardiometabolic outcomes, safety and tolerability outcomes, and treatment persistence. Extracted data were cross-checked, and discrepancies were resolved by reference to the source publication. Where a study reported results under more than one estimand or analysis population, the primary prespecified analysis was extracted. No study authors were contacted, and no data were imputed; outcomes not reported in a given study are recorded as not reported rather than inferred.

Risk-of-Bias Assessment

Risk of bias was assessed independently by two reviewers using design-appropriate validated instruments. The single randomised trial was appraised with the revised Cochrane risk-of-bias tool for randomised trials (RoB 2) across the domains of randomisation process, deviations from intended interventions, missing outcome data, measurement of the outcome, and selection of the reported result, with an overall judgement of low risk, some concerns, or high risk [12]. The 10 non-randomised comparative cohorts were appraised with the Risk Of Bias In Non-randomised Studies - of Interventions (ROBINS-I) tool across the domains of confounding, selection of participants, classification of interventions, deviations from intended interventions, missing data, measurement of outcomes, and selection of the reported result, with domain and overall judgements of low, moderate, serious, or critical risk of bias [13]. Consistent with the ROBINS-I algorithm, the overall judgement for each study was taken as the most severe judgement assigned to any single domain. Disagreements were resolved by consensus.

Data Synthesis

A structured narrative synthesis was performed in accordance with PRISMA 2020 guidance for reviews without meta-analysis [11]. Findings were tabulated and grouped by outcome domain: continuous weight change, categorical weight-reduction thresholds, cardiometabolic parameters, incident disease outcomes, and safety and tolerability. Results were interpreted with explicit attention to study design, risk-of-bias judgement, and the distinction between efficacy under trial conditions and effectiveness in routine care.

The decision not to undertake meta-analysis was prespecified as conditional on the characteristics of the retrieved evidence and was taken for five specific reasons rather than as a default. First, the evidence base comprises a single randomised trial and 10 non-randomised cohorts; pooling randomised and non-randomised estimates in one summary effect is discouraged, because the resulting figure has no coherent causal interpretation and the non-randomised studies, being far larger, would dominate the weighting and effectively determine a result nominally presented as including randomised evidence. Second, the studies differ fundamentally in the estimand addressed, encompassing on-treatment analyses, persistent-user analyses conditioned on one year of continuous therapy, modified intention-to-treat analyses, and both treatment-regimen and efficacy estimands within the randomised trial; these quantities answer different clinical questions and are not interchangeable inputs to a common effect size. Third, follow-up ranged from six months to 72 weeks, and one included cohort demonstrated that the between-group difference approximately triples between three and 12 months, so pooling across durations would average over a systematically time-dependent effect. Fourth, the comparator was not consistent, since some studies used the formulations and doses licensed for glycaemic management and others those licensed for weight management, and one study performed no between-group statistical testing and reported no variance estimates, making it uncombinable. Fifth, two cohorts drew on the same underlying electronic health record platform, so the assumption of independent samples that underpins standard meta-analytic variance estimation may not hold. Because no pooling was performed, statistical measures of heterogeneity, such as I-squared and tau-squared, and formal tests for small-study effects, such as Egger's test, are not estimable and are therefore not reported; heterogeneity is instead characterised qualitatively across the dimensions set out above.

Results

Study Selection

**Figure 1:**
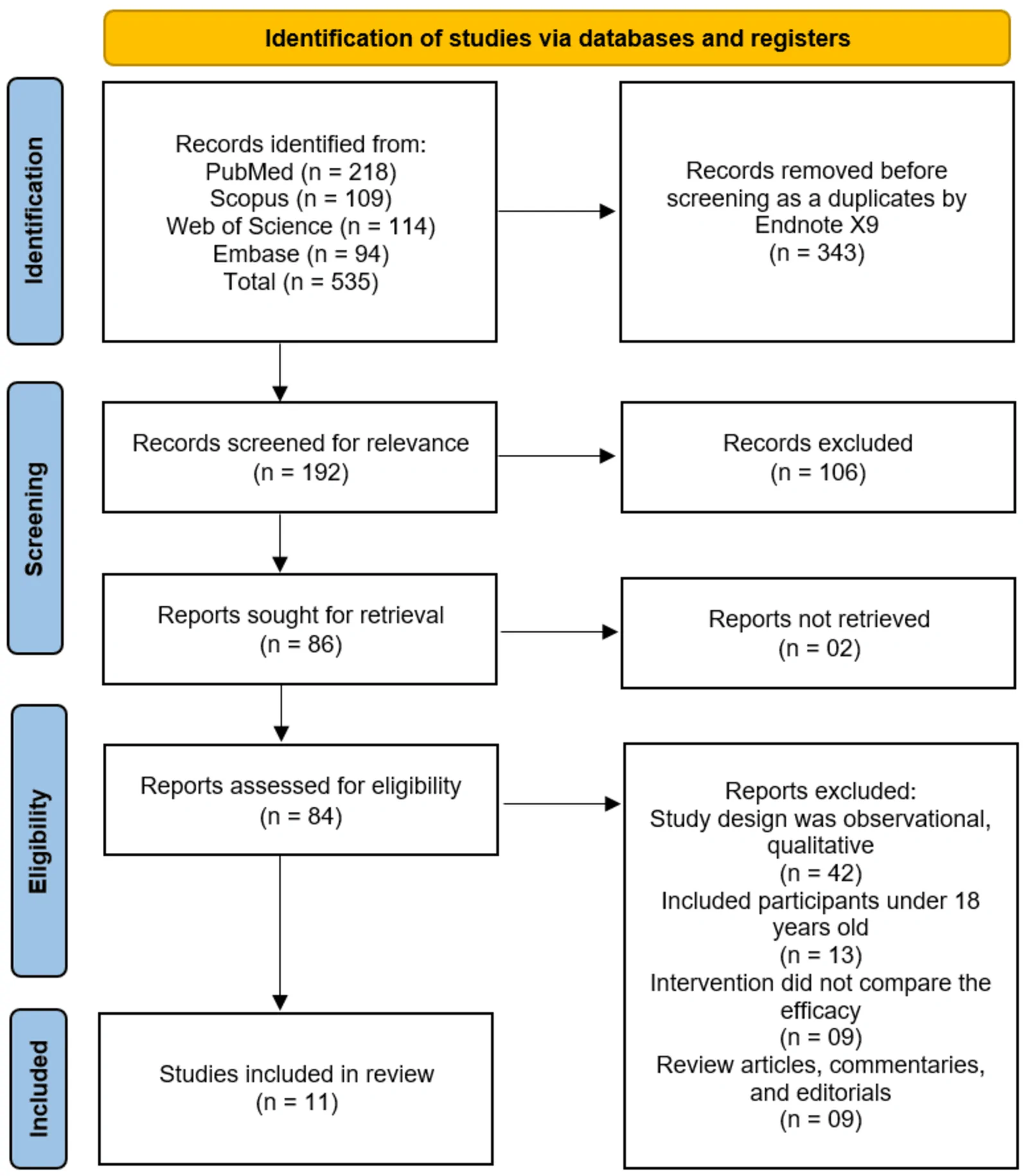
PRISMA flowchart of studies selection process

The database searches identified 535 records: 218 from PubMed, 114 from Web of Science, 109 from Scopus, and 94 from Embase. After 343 duplicates were removed in EndNote X9, 192 records were screened for relevance, and 106 were excluded. Of the 86 reports sought for retrieval, two could not be obtained, leaving 84 assessed in full text. Seventy-three reports were excluded: 42 because the study design was ineligible, 13 because participants under 18 years of age were included, nine because the intervention did not compare the efficacy of the two agents, and nine because they were review articles, commentaries, or editorials. Eleven studies published between 2024 and 2026 met all eligibility criteria and were included in the narrative synthesis, together contributing approximately 62,700 analysed participants. The selection process is summarised in Figure 1.

Characteristics of Included Studies

The characteristics of the 11 included studies are summarised in Table 3. One study was a randomised trial, and 10 were retrospective observational cohorts. Rodriguez et al. analysed a propensity-score-matched cohort of 18,386 adults with overweight or obesity drawn from a multi-health-system electronic health record collective in the United States, approximately half of whom had type 2 diabetes [14]. Jamal et al. reported a single-institution Kuwaiti cohort of 115 adults treated for weight recurrence after sleeve gastrectomy [15]. Anson et al. used the international TriNetX federated network to construct propensity-matched cohorts of 13,846 adults living with obesity without type 2 diabetes and 8,446 adults with pre-existing type 2 diabetes [16]. Trinh et al. described a single-centre ambulatory care cohort of 416 patients in Nebraska, United States [17]. Aronne et al. reported SURMOUNT-5, a 72-week phase 3b, multicentre, open-label, randomised, active-controlled trial conducted at 32 sites in the United States and Puerto Rico in 751 adults with obesity but without type 2 diabetes [18]. Gasoyan et al. studied 7,881 adults with overweight or obesity without type 2 diabetes within a large health system in Ohio and Florida, with treatment discontinuation as the organising exposure [19]. Ng et al. reported the SHAPE study, a descriptive claims-based cohort of 9,916 persistent users without type 2 diabetes in the United States [20]. Shil et al. compared 100 adults with obesity across three tertiary centres in Bangladesh [21]. Al Ozairi et al. compared tirzepatide, semaglutide, and liraglutide over 12 months in 250 adults with a BMI of at least 27 kg/m² and type 1 diabetes in Kuwait [22]. le Roux et al. analysed 2,396 treatment-adherent adults with obesity without diabetes who initiated the obesity-labelled formulations of either agent in United States routine care [23]. Cetiner reported a single-centre Turkish cohort of 169 adults with overweight or obesity without diabetes treated for at least 36 weeks [24]. Mean baseline body weight, where reported, ranged from approximately 85 kg to 113 kg and mean baseline BMI from approximately 31 kg/m² to 39.7 kg/m², and women constituted the majority of participants in every cohort (54.8% to 87%).

**Table 3:** Characteristics of the 11 included studies comparing tirzepatide with semaglutide in adults with overweight or obesity aOR: adjusted odds ratio; BMI: body mass index; GLP-1: glucagon-like peptide-1; HbA1c: glycated haemoglobin; HDL: high-density lipoprotein; HOMA-IR: homeostatic model assessment of insulin resistance; HR: hazard ratio; LDL: low-density lipoprotein; T2D: type 2 diabetes

Author (year)	Country/setting	Study design	Population	Sample size	Intervention	Comparator	Follow-up and outcomes	Key findings
Rodriguez et al. (2024) [14]	USA; multi-health-system electronic health records	Retrospective propensity-score-matched cohort	Adults with overweight or obesity, GLP-1 receptor agonist naive; 52% with type 2 diabetes; mean age 52 years; 70.5% women	18,386 matched (9,193 vs 9,192)	Tirzepatide (T2D-labelled)	Semaglutide (T2D-labelled)	12 months. Time to 5%, 10% and 15% loss; weight change at 3, 6 and 12 months; gastrointestinal adverse events	Greater loss with tirzepatide at all timepoints (-6.9 percentage points at 12 months). HR for at least 15% loss 3.24 (2.91-3.61). Gastrointestinal adverse event rates similar.
Jamal et al. (2024) [15]	Kuwait; single tertiary institution	Retrospective cohort	Adults with weight recurrence after sleeve gastrectomy; mean age 38.8 years; 80.9% women; mean BMI 35.1 kg/m²	115 (45 vs 70)	Tirzepatide	Semaglutide	6 months. Absolute and percentage weight change	Mean 6-month loss 15.5% with tirzepatide versus 10.3% with semaglutide (both P < 0.05). Groups differed at baseline and no adjustment was applied.
Anson et al. (2024) [16]	International; TriNetX federated network	Retrospective propensity-score-matched cohort	Two matched cohorts: adults with obesity without type 2 diabetes, and adults with pre-existing type 2 diabetes	22,292 (13,846 without and 8,446 with T2D)	Tirzepatide	Semaglutide	12 months. Incident type 2 diabetes; weight change; composite cardiovascular outcome; mortality	Greater weight loss (-7.7 versus -4.8 kg) and lower incidence of type 2 diabetes (HR 0.73) with tirzepatide. In the diabetes cohort, lower composite cardiovascular outcome (HR 0.54) and all-cause mortality (HR 0.33).
Trinh et al. (2025) [17]	USA (Nebraska); single academic ambulatory clinic	Retrospective cohort (brief report)	Adults with overweight or obesity, with and without diabetes, in routine ambulatory care	416 (60 vs 356)	Tirzepatide	Semaglutide	6 months. Weight change; more than 5% and 10% loss; subgroup analysis by diabetes status	Mean loss 6.6 versus 3.1 kg (P < 0.001). Benefit confined to patients without diabetes (additional 3.6%, P < 0.001); no significant difference in those with diabetes (P = 0.70).
Aronne et al. (2025) [18] (SURMOUNT-5)	USA and Puerto Rico; 32 trial sites	Phase 3b open-label randomised active-controlled trial (NCT05822830)	Adults with BMI at least 30 kg/m², or at least 27 kg/m² with an obesity-related complication, without type 2 diabetes; mean age 44.7 years; 64.7% women; mean BMI 39.4 kg/m²	751 randomised (750 treated)	Tirzepatide 10 or 15 mg weekly	Semaglutide 1.7 or 2.4 mg weekly	72 weeks. Percentage weight change (primary); categorical thresholds; waist circumference; cardiometabolic parameters; adverse events	Tirzepatide superior for weight (-20.2% versus -13.7%; P < 0.001) and waist circumference (-18.4 versus -13.0 cm), with higher attainment of all thresholds and greater improvement in blood pressure, HbA1c, insulin and lipids. Gastrointestinal events predominated in both arms.
Gasoyan et al. (2025) [19]	USA (Ohio and Florida); integrated health system	Retrospective cohort; discontinuation as organising exposure	Adults with overweight or obesity without type 2 diabetes; mean age 51.3 years; 75.2% women; mean BMI 39.7 kg/m²	7,881 (1,772 vs 6,109)	Tirzepatide	Semaglutide	12 months. Percentage weight change; at least 10% loss; HbA1c in prediabetes; discontinuation and dose	Among non-discontinuers, loss was 15.3% with tirzepatide versus 10.9% with semaglutide. Tirzepatide, persistence, higher maintenance dose and female sex independently predicted at least 10% loss.
Ng et al. (2025) [20] (SHAPE)	USA; national claims and electronic medical record database	Retrospective descriptive cohort; no between-group testing	Adults with overweight or obesity without type 2 diabetes, persistent on therapy for 1 year; mean age 47.8 and 49.5 years; about 79% women	9,916 (3,122 vs 6,794)	Tirzepatide, any dose	Semaglutide 2.4 mg	12 months. Weight change; at least 5% loss; maximum dose attained	Loss of -17.2 kg (-16.5%) versus -14.6 kg (-14.1%) at 1 year; descriptive only. Maximum labelled dose reached by 25.9% versus 83.5%.
Shil et al. (2025) [21]	Bangladesh; three tertiary centres	Retrospective observational study	Adults with obesity treated for at least 3 months; median age 27 years; 87% women; baseline BMI higher with tirzepatide (36.6 versus 32.3 kg/m²)	100 (58 vs 42)	Tirzepatide	Semaglutide	Up to 12 months. Weight change; categorical thresholds; lifestyle adherence; adverse events	Greater loss with tirzepatide (8.53 versus 6.85 kg; P = 0.033). Lifestyle intervention enhanced reduction, most notably with tirzepatide. Gastrointestinal events more frequent with tirzepatide; serious events rare.
Al Ozairi et al. (2026) [22]	Kuwait; specialist diabetes institute	Observational cohort with four treatment groups	Adults with BMI at least 27 kg/m² and type 1 diabetes; mean BMI 31.6 kg/m²; 54.8% women	250 (35 vs 36; liraglutide 97; usual care 82)	Tirzepatide	Semaglutide (also liraglutide, usual care)	12 months. Percentage weight change; HbA1c; lipids; renal and liver markers	Weight loss 10.9% (tirzepatide), 9.9% (semaglutide) and 7.1% (liraglutide), all P < 0.001. HbA1c fell most with tirzepatide (-0.65%). LDL cholesterol fell with semaglutide and liraglutide only.
le Roux et al. (2026) [23]	USA; 30 health systems across all 50 states	Retrospective propensity-score-weighted cohort with sensitivity analyses	Adults with obesity without diabetes initiating obesity-labelled formulations and adherent (proportion of days covered at least 80%); mean age 48.4 and 49.1 years; mean BMI 38.3 kg/m²	2,396 (1,003 vs 1,393)	Tirzepatide (obesity-labelled)	Semaglutide (obesity-labelled)	6 months. Percentage and absolute weight change; 5-20% thresholds; BMI; blood pressure; HbA1c; lipids	Adjusted loss -11.15% versus -8.83% (difference -2.32 percentage points). All thresholds attained more often with tirzepatide (adjusted OR 2.03-3.23), with greater improvement in blood pressure and HbA1c. Advantage persisted despite lower dose attainment.
Cetiner (2026) [24]	Turkiye (Istanbul); single private clinic	Retrospective observational study with three treatment groups	Adults aged 18-75 years with overweight or obesity without diabetes treated for at least 36 weeks; 58% women	169 (59 vs 55; liraglutide 55)	Tirzepatide	Semaglutide (also liraglutide)	36 weeks. Percentage weight change; waist circumference; lipids; liver and pancreatic enzymes; HOMA-IR; adverse events	Greatest weight loss with tirzepatide (P < 0.01 versus both comparators), with the 10% and 20% thresholds reached more often and greater triglyceride reduction (P = 0.009). No confirmed pancreatitis.

Comparative Weight Reduction

Comparative weight outcomes are presented in Table 4. Tirzepatide produced greater weight reduction than semaglutide in every study in which the comparison was formally tested, and the direction of effect was consistent across randomised and non-randomised designs, across geographies, and across diabetes strata. The largest absolute difference was observed in the randomised setting: in SURMOUNT-5, mean percentage change in body weight at week 72 was -20.2% with tirzepatide compared with -13.7% with semaglutide, a difference of 6.5 percentage points (P < 0.001) under the treatment-regimen estimand, and -21.6% versus -15.4% under the efficacy estimand [18]. Differences observed in routine care were consistently smaller. At six months, le Roux et al. reported adjusted percentage weight changes of -11.15% (95% CI -11.82 to -10.48) with tirzepatide and -8.83% (95% CI -9.33 to -8.33) with semaglutide, an adjusted difference of -2.32 percentage points (95% CI -3.17 to -1.48), corresponding to -12.36 kg versus -9.45 kg and a BMI reduction of -5.12 versus -3.86 kg/m² [23]. Rodriguez et al. reported adjusted on-treatment differences favouring tirzepatide of -2.4 percentage points at three months (95% CI -2.5 to -2.2), -4.3 percentage points at six months (95% CI -4.7 to -4.0), and -6.9 percentage points at 12 months (95% CI -7.9 to -5.8) [14]. Among patients who did not discontinue therapy within one year, Gasoyan et al. observed a mean reduction of 15.3% (95% CI 14.6 to 16.0) with tirzepatide versus 10.9% (95% CI 10.5 to 11.2) with semaglutide [19]. In the propensity-matched cohort without type 2 diabetes reported by Anson et al., weight loss was -7.7 kg (95% CI -6.8 to -8.5) with tirzepatide and -4.8 kg (95% CI -3.9 to -5.6) with semaglutide [16]. Smaller cohorts were directionally concordant: 15.5% versus 10.3% at six months after sleeve gastrectomy [15]; 6.6 kg versus 3.1 kg in ambulatory care, with an additional 3.6% weight loss attributable to tirzepatide among patients without diabetes (95% CI -5.5 to -1.8; P < 0.001) but no significant difference among those with diabetes [17]; 8.53 kg versus 6.85 kg in Bangladesh (P = 0.033) [21]; and 10.9% versus 9.9% over 12 months in adults with type 1 diabetes [22]. The Turkish cohort found significantly greater mean absolute weight loss with tirzepatide than with either semaglutide or liraglutide at week 36 (P < 0.01 for both comparisons) [24]. The single exception to formal statistical superiority was the SHAPE study, which was explicitly descriptive and performed no between-group testing; it nonetheless reported one-year weight loss of -17.2 kg (-16.5%) with tirzepatide and -14.6 kg (-14.1%) with semaglutide 2.4 mg [20].

**Table 4:** Comparative weight, cardiometabolic and safety outcomes reported by the included studies Outcomes recorded as not reported were not presented for both treatment arms in the source publication and have not been inferred. aOR: adjusted odds ratio; CI: confidence interval; HbA1c: glycated haemoglobin; HDL: high-density lipoprotein; HOMA-IR: homeostatic model assessment of insulin resistance; HR: hazard ratio; LDL: low-density lipoprotein; pp: percentage points

Study	Follow-up	Weight change: tirzepatide	Weight change: semaglutide	Between-group difference	Categorical thresholds	Cardiometabolic and incident disease	Safety and treatment behaviour
Rodriguez et al. [14]	3, 6, 12 months	Greater reduction at every timepoint	Smaller reduction at every timepoint	-2.4 pp (3 months); -4.3 pp (6 months); -6.9 pp (12 months), 95% CI -7.9 to -5.8	HR 1.76 (at least 5%); 2.54 (at least 10%); 3.24 (at least 15%)	Not reported	Similar rates of moderate-to-severe gastrointestinal events; discontinuation common in both groups
Jamal et al. [15]	6 months	15.5% (100.2 to 87.6 kg)	10.3% (90.1 to 81.0 kg)	About 5.2 pp; unadjusted, no formal test	Not reported	Not reported	Not reported
Anson et al. [16]	12 months	-7.7 kg (95% CI -6.8 to -8.5)	-4.8 kg (95% CI -3.9 to -5.6)	About 2.9 kg favouring tirzepatide	Not reported	Incident type 2 diabetes HR 0.73 (0.58-0.92). Diabetes cohort: composite cardiovascular HR 0.54; cerebral infarction HR 0.45; mortality HR 0.33	Not reported
Trinh et al. [17]	6 months	-6.6 kg	-3.1 kg	P < 0.001 overall; +3.6% in patients without diabetes (P < 0.001); no difference with diabetes (P = 0.70)	More than 5% loss: 48.6% versus 26.0%	Not reported	Not reported; no persistence requirement and dose not analysed
Aronne et al. [18]	72 weeks	-20.2% (efficacy estimand -21.6%)	-13.7% (efficacy estimand -15.4%)	6.5 pp (P < 0.001); waist circumference -18.4 versus -13.0 cm	Higher attainment of at least 10%, 15%, 20% and 25%	Greater improvement in systolic and diastolic blood pressure, HbA1c, fasting insulin, triglycerides and HDL cholesterol	Gastrointestinal events most common in both arms, predominantly mild to moderate
Gasoyan et al. [19]	12 months	15.3% (non-discontinuers)	10.9% (non-discontinuers)	About 4.4 pp; whole-cohort mean 8.7%	Tirzepatide independently predicted at least 10% loss, with persistence, higher dose and female sex	HbA1c -0.4% versus -0.3% in baseline prediabetes	Early discontinuation 16.4% versus 21.6%; late 34.1% versus 31.4%; 80.8% on low maintenance dosages
Ng et al. [20]	12 months	-17.2 kg (-16.5%)	-14.6 kg (-14.1%)	No between-group testing (descriptive design)	At least 5% loss: 86.2% versus 84.1%	Not reported	Maximum dose reached by 25.9% versus 83.5%; 1-year persistence required
Shil et al. [21]	Up to 12 months	-8.53 kg	-6.85 kg	P = 0.033; baseline BMI higher with tirzepatide	At least 5% in 89 of 100 and at least 10% in 33 of 100 overall, more often with tirzepatide	Not reported	Gastrointestinal events more frequent with tirzepatide; serious events rare
Al Ozairi et al. [22]	12 months	-10.9% (P < 0.001)	-9.9% (P < 0.001)	About 1.0 pp; liraglutide -7.1%	Not reported	HbA1c -0.65%, -0.33% and -0.23% for tirzepatide, semaglutide and liraglutide; LDL cholesterol fell with semaglutide and liraglutide only	Dose-dependent weight effects for both agents
le Roux et al. [23]	6 months	-11.15%; -12.36 kg; BMI -5.12 kg/m²	-8.83%; -9.45 kg; BMI -3.86 kg/m²	-2.32 pp (95% CI -3.17 to -1.48); -2.91 kg; BMI -1.25 kg/m²	At least 5%: 85.7% versus 75.2% (aOR 2.03); 10%: 59.0% versus 38.0% (2.46); 15%: 31.2% versus 14.0% (2.88); 20%: 11.3% versus 3.8% (3.23)	Diastolic blood pressure -3.51 versus -1.99 mmHg; systolic -7.32 versus -5.90 mmHg; HbA1c -0.52% versus -0.37%; lipids similar	Higher doses reached by 42.4% versus 67.7%; adherence required for the primary cohort
Cetiner [24]	36 weeks	Greatest reduction of the three agents	Significant within-group reduction, smaller than tirzepatide	P < 0.01 versus semaglutide and versus liraglutide	At least 5% similar across groups; 10% and 20% reached more often with tirzepatide	Waist circumference reduced in all groups; greater triglyceride reduction with tirzepatide (P = 0.009); HOMA-IR and liver enzymes improved in all groups	Gastrointestinal events common, particularly with liraglutide; lipase rose within all groups; no confirmed pancreatitis

Categorical Weight-Reduction Thresholds and Other Anthropometric Outcomes

The comparative advantage of tirzepatide was consistently more pronounced at more demanding weight-reduction thresholds. Rodriguez et al. reported hazard ratios for achieving weight loss of at least 5%, 10%, and 15% of 1.76 (95% CI 1.68 to 1.84), 2.54 (95% CI 2.37 to 2.73), and 3.24 (95% CI 2.91 to 3.61), respectively [14]. le Roux et al. observed that 85.7% versus 75.2% of patients achieved at least 5% reduction (adjusted OR 2.03, 95% CI 1.63 to 2.54), 59.0% versus 38.0% at least 10% (adjusted OR 2.46, 95% CI 2.06 to 2.93), 31.2% versus 14.0% at least 15% (adjusted OR 2.88, 95% CI 2.32 to 3.57), and 11.3% versus 3.8% at least 20% (adjusted OR 3.23, 95% CI 2.29 to 4.55), all P < 0.0001; a shift of at least one BMI category was achieved by 68.8% versus 54.8% (adjusted OR 1.92, 95% CI 1.59 to 2.32) [23]. In SURMOUNT-5, higher proportions of tirzepatide-treated participants reached each of the 10%, 15%, 20%, and 25% thresholds, and mean waist circumference fell by 18.4 cm versus 13.0 cm [18]. Gasoyan et al. identified tirzepatide, non-discontinuation or late rather than early discontinuation, higher maintenance dosage, and female sex as independent predictors of achieving at least 10% weight reduction at one year [19]. In the Turkish cohort, achievement of at least 5% reduction was similar across agents, whereas the 10% and 20% thresholds were reached more frequently with tirzepatide [24], and in the Bangladeshi cohort, 89 of 100 participants achieved at least 5% and 33 achieved at least 10% reduction, with higher proportions in the tirzepatide group [21]. In SHAPE, 86.2% of tirzepatide-treated and 84.1% of semaglutide-treated persistent users achieved at least 5% weight loss [20].

Cardiometabolic and Incident-Disease Outcomes

In SURMOUNT-5, tirzepatide produced significantly greater improvements than semaglutide at 72 weeks in systolic and diastolic blood pressure, HbA1c, fasting insulin, triglycerides, and high-density lipoprotein cholesterol [18]. le Roux et al. reported greater reductions with tirzepatide in diastolic blood pressure (-3.51 versus -1.99 mmHg; adjusted difference -1.52, 95% CI -2.29 to -0.75), systolic blood pressure (-7.32 versus -5.90 mmHg; adjusted difference -1.42, 95% CI -2.50 to -0.35), and HbA1c (-0.52% versus -0.37%; adjusted difference -0.15, 95% CI -0.24 to -0.05), with no significant between-group differences in lipid parameters [23]. Anson et al. found that tirzepatide was associated with a lower risk of new-onset type 2 diabetes among adults living with obesity (HR 0.73, 95% CI 0.58 to 0.92) and, in the separate cohort with pre-existing type 2 diabetes, with lower risks of the composite cardiovascular outcome (HR 0.54, 95% CI 0.38 to 0.76), cerebral infarction (HR 0.45, 95% CI 0.24 to 0.84), and all-cause mortality (HR 0.33, 95% CI 0.15 to 0.73) [16]. Among participants with prediabetes at baseline, Gasoyan et al. reported one-year HbA1c reductions of 0.4% with tirzepatide and 0.3% with semaglutide [19]. In adults with type 1 diabetes, HbA1c fell by 0.65% with tirzepatide, 0.33% with semaglutide, and 0.23% with liraglutide [22]. The Turkish cohort reported significant within-group reductions in waist circumference, triglycerides, and homeostatic model assessment of insulin resistance across all three agents, with a significantly greater triglyceride reduction in the tirzepatide group (P = 0.009) and similar improvement in liver enzymes [24].

Safety, Tolerability, Dose Attainment, and Persistence

Safety and tolerability were reported inconsistently, and only four studies presented comparative adverse event data. In SURMOUNT-5, gastrointestinal events were the most common adverse events in both treatment groups and were predominantly mild to moderate in severity [18]. Rodriguez et al., using a prespecified set of moderate-to-severe gastrointestinal adverse events including bowel obstruction, cholecystitis, cholelithiasis, gastroenteritis, gastroparesis, and pancreatitis, found similar rates between the two agents [14]. Shil et al. reported that gastrointestinal adverse events were the most frequent events overall and occurred more often with tirzepatide, while serious events were rare [21]. Cetiner reported that gastrointestinal adverse events were common across all three incretin agents and particularly frequent with liraglutide, that serum lipase rose significantly within all treatment groups without significant between-group differences, and that no clinically confirmed case of pancreatitis occurred [24]. Treatment persistence emerged as an important effect modifier: Gasoyan et al. observed that 21.6% of semaglutide-treated and 16.4% of tirzepatide-treated patients discontinued within three months and a further 31.4% and 34.1% respectively between three and 12 months, and that 80.8% of the cohort remained on low maintenance dosages, with an overall one-year weight reduction of 8.7% that fell to 3.6% among early discontinuers and rose to 11.9% among those who did not discontinue [19]. Dose attainment differed markedly between agents in routine care: 83.5% of semaglutide-treated but only 25.9% of tirzepatide-treated persistent users reached the maximum labelled dose in SHAPE [20], and le Roux et al. found that 67.7% of semaglutide-treated compared with 42.4% of tirzepatide-treated patients reached the higher available maintenance doses, yet the weight advantage of tirzepatide persisted [23].

Risk of Bias in Included Studies

**Figure 2:**
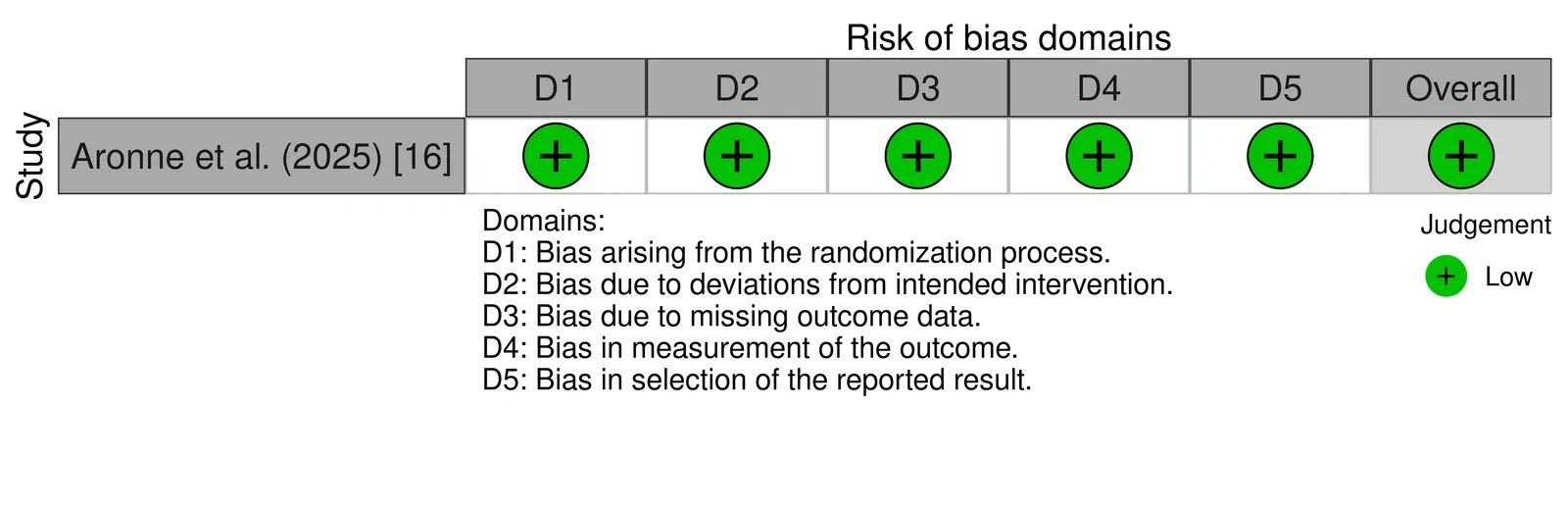
Risk of bias using Cochrane RoB 2

**Figure 3:**
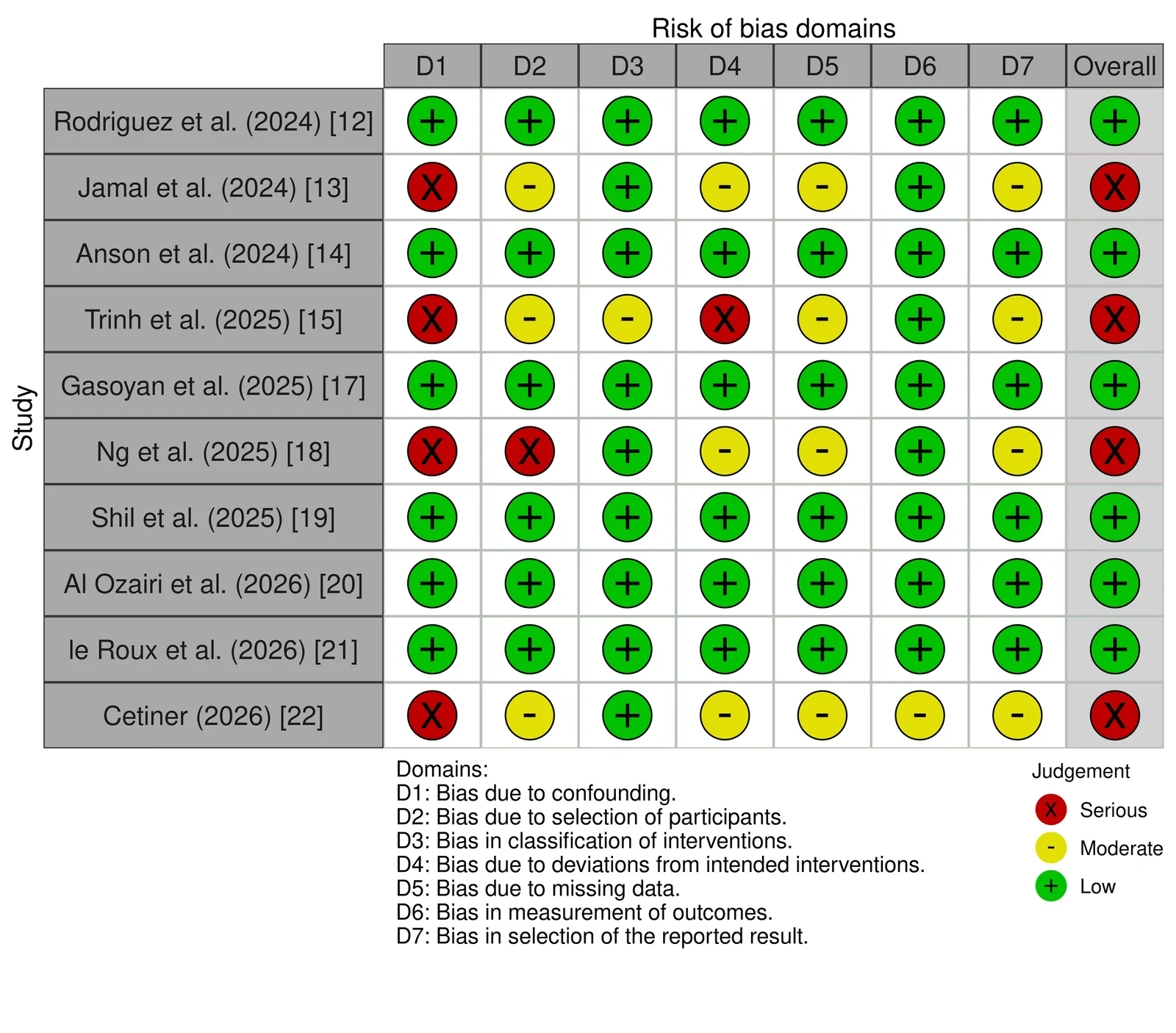
Non-randomised comparative studies assessed using the Risk Of Bias In Non-randomised Studies - of Interventions (ROBINS-I) tool

Risk-of-bias judgements are presented in Figures 2-3. SURMOUNT-5 was judged at low risk of bias in all five RoB 2 domains and overall: randomisation and allocation concealment were adequate, missing outcome data were balanced across arms and appropriately handled, the primary anthropometric endpoint was objectively measured and unlikely to be influenced by knowledge of assignment, and the reported analysis followed the prespecified protocol [18]. Among the 10 non-randomised studies, six were judged at low overall risk of bias [14,16,19,21-23] and four at serious risk [15,17,20,24]. The low-risk cohorts addressed confounding adequately, applied consistent intervention definitions, and reported prespecified analyses with near-complete ascertainment of objectively recorded anthropometric outcomes; three balanced treatment groups using propensity-score matching or weighting across broad covariate sets [14,16,23], one of which supplemented this with frequentist model averaging, principal stratification, and E-value sensitivity analysis [23]. The four studies judged at serious risk were limited by unadjusted comparison of non-equivalent treatment groups, small and markedly unbalanced arm sizes, or an explicitly descriptive design without between-group statistical testing; confounding drove the overall judgement in each, with selection bias from a one-year persistence requirement contributing in one [20].

Discussion

This systematic review identified 11 original comparative studies, encompassing approximately 62,700 analysed participants, that directly compared subcutaneous tirzepatide with subcutaneous semaglutide in adults with overweight or obesity. The central finding is one of striking consistency: in every study that formally tested the comparison, tirzepatide produced greater weight reduction than semaglutide, and this held across a randomised trial and 10 observational cohorts, across five countries and an international federated network, across populations with and without type 2 diabetes, in adults with type 1 diabetes, and in patients experiencing weight recurrence after sleeve gastrectomy. Such uniformity of direction across designs subject to very different biases is itself informative, because the threats to validity in a 72-week open-label randomised trial bear little resemblance to those in a claims-based analysis of persistent users. Consistency of direction should not, however, be mistaken for equivalence of magnitude. The between-group difference varied by a factor of roughly three across the evidence base, from 6.5 percentage points at 72 weeks in SURMOUNT-5 [18] to approximately 2.3 percentage points at six months in a propensity-weighted analysis of routine United States care [23], and the interpretation of that gradient is where the clinical value of this synthesis principally lies.

Three structural features of the included studies explain much of this gradient. Follow-up duration is the most obvious: the divergence between agents widens progressively over time, as demonstrated within a single cohort by Rodriguez et al., whose adjusted difference grew from 2.4 percentage points at three months to 4.3 at six months and 6.9 at 12 months [14], a trajectory closely mirroring the SURMOUNT-5 interim data. Studies with six-month horizons therefore necessarily understate the eventual separation. Dose attainment is the second and more counterintuitive factor. In routine care, patients receiving semaglutide reach the maximum labelled dose far more often than patients receiving tirzepatide: 83.5% versus 25.9% in SHAPE [20] and 67.7% versus 42.4% for the higher maintenance dose bands in the Truveta analysis [23]. That the tirzepatide advantage persists despite this systematic disadvantage in relative dose intensity strengthens rather than weakens the inference of superior pharmacological efficacy, and suggests the real-world gap would widen further if titration practices converged. Third, adherence and persistence exert a powerful influence largely independent of drug choice. Gasoyan et al. found that overall one-year weight reduction of 8.7% fell to 3.6% among patients discontinuing within three months but reached 11.9% among those who persisted, and that persistence, maintenance dose, drug choice, and sex were all independent predictors of achieving at least 10% reduction [19]. The practical corollary is that a clinician choosing between these agents is making a decision whose expected benefit is smaller in magnitude than the decision about whether the patient can be supported to remain on treatment at an adequate dose.

These findings align with, and materially extend, the existing comparative literature. A prior systematic review and meta-analysis of direct comparative studies searched to April 2025 identified seven studies with 28,980 participants and reported a pooled standardised mean difference of 0.76 (95% CI 0.53 to 1.00) favouring tirzepatide, together with substantially higher odds of achieving at least 10% weight loss, but noted heterogeneity exceeding 90% and could not assess comparative safety because only two studies reported adverse events in both arms [25]. The present review captures four additional eligible studies published subsequently, including two 2026 cohorts from Turkiye and the United States and a comparison in adults with type 1 diabetes, thereby broadening the geographical and clinical range of the evidence considerably. It also reaches a different methodological conclusion. Because the included studies differ in comparator formulation, licensed indication, follow-up duration, analysis population, and estimand, and because four of the 10 non-randomised studies were judged at serious risk of bias, quantitative pooling was judged inappropriate; pooling heterogeneous observational estimates alongside a single randomised trial risks generating a spuriously precise summary effect dominated by the largest datasets, and the very high heterogeneity reported previously supports that caution. Interpreted against the pre-existing indirect evidence, the direct data are broadly concordant with what cross-trial comparison of STEP 1 [4] and SURMOUNT-1 [5] would have predicted, and with the SURPASS-2 finding in type 2 diabetes [10], but they now place that conclusion on a far more secure footing, since SURMOUNT-5 compared both agents at their maximum tolerated obesity-licensed doses within a single randomised population.

The distribution of benefit across categorical thresholds may be more clinically consequential than the difference in mean weight change. The relative advantage of tirzepatide increased monotonically with the stringency of the threshold in every study reporting them, with odds or hazard ratios rising from approximately 1.8 to 2.0 at the 5% threshold to approximately 2.9 to 3.2 at the 15% and 20% thresholds [14,23]. Because improvements in obstructive sleep apnoea, hepatic steatosis, glycaemia, blood pressure, and remission of type 2 diabetes are broadly graded by the degree of weight loss, with several requiring reductions well beyond 10%, a therapy that approximately doubles the proportion of patients reaching 15% reduction may deliver clinical benefit disproportionate to a mean difference of two or three percentage points. Framing the comparison in terms of the number needed to treat to achieve a clinically decisive threshold is therefore likely to be more informative for shared decision-making than reporting mean differences alone.

Evidence on outcomes beyond weight remains markedly asymmetrical between the two agents, and this asymmetry is arguably the most important unresolved issue in the field. Semaglutide has a definitive randomised cardiovascular outcome trial in the obesity population: SELECT demonstrated a 20% reduction in major adverse cardiovascular events in adults with overweight or obesity and established cardiovascular disease without diabetes [8]. No equivalent randomised cardiovascular outcome trial of tirzepatide in obesity without diabetes has been reported. The included studies provide only observational signals in this direction. Anson et al. reported a lower incidence of new-onset type 2 diabetes with tirzepatide, together with lower rates of a composite cardiovascular outcome, cerebral infarction, and all-cause mortality in a separate cohort with pre-existing type 2 diabetes [16]; effect sizes of that magnitude in a non-randomised design should be regarded as hypothesis-generating, since residual confounding by indication and healthy-adherer effects can readily produce apparent mortality reductions of that order. Contemporary evidence outside this review reinforces that caution: a large clinical-practice cohort study comparing cardiovascular outcomes with semaglutide and tirzepatide in type 2 diabetes found broadly comparable event rates [26], and in the randomised SURPASS-CVOT trial tirzepatide was non-inferior, but not clearly superior, to dulaglutide for major adverse cardiovascular events [27]. The current evidence therefore supports greater weight and cardiometabolic risk-factor improvement with tirzepatide while leaving open whether that translates into superior hard cardiovascular outcomes.

Comparative safety data are the weakest component of the evidence base, and this review should not be read as demonstrating equivalent tolerability. Only four of the 11 included studies reported adverse events for both arms. Gastrointestinal events predominated with both agents and were mostly mild to moderate in the randomised setting [18]. Rodriguez et al. found similar rates of moderate-to-severe gastrointestinal events between agents in a large matched cohort [14], whereas Shil et al. reported more frequent gastrointestinal events with tirzepatide in a small cohort with significantly higher baseline BMI in the tirzepatide arm [21], and Cetiner observed common gastrointestinal events across all incretin agents, most frequently with liraglutide, with significant within-group increases in serum lipase but no confirmed pancreatitis [24]. Adverse event ascertainment in electronic health record and claims data is systematically incomplete for symptomatic, self-limiting events that do not prompt coded encounters, and the reliance in several studies on persistent users introduces a survivor effect that preferentially excludes patients who discontinued because of intolerance. The observation that early discontinuation was somewhat more common with semaglutide than tirzepatide [19] should therefore be interpreted with care, since it may reflect access, cost, and supply factors as much as tolerability. Rare but clinically important events, including pancreatitis, biliary disease, gastroparesis, and any difference in loss of lean mass, cannot be adequately compared from the current evidence.

Several knowledge gaps follow directly from these findings. No randomised comparison extends beyond 72 weeks, yet obesity pharmacotherapy is a long-term intervention, and SURMOUNT-4 established that discontinuation is followed by substantial weight regain [9]; whether the tirzepatide advantage is maintained, widens, or narrows over three to five years is unknown. There is no head-to-head randomised comparison in adults with obesity and type 2 diabetes, a population in which weight loss is consistently attenuated and in which one included study found no significant between-agent difference [17], and in which SURMOUNT-2 indicates a materially different response profile [7]. The evidence also remains overwhelmingly North American: only four included studies were conducted elsewhere, three of them in single centres with fewer than 200 participants [15,21,22,24], leaving South Asian, East Asian, African, and Latin American populations largely unrepresented despite well-described differences in body composition and cardiometabolic risk at any given BMI. No included study reported body composition, so the relative preservation of lean mass, bone mineral density, and physical function is unknown, and comparative data in adolescents, older adults, and pregnancy planning are absent. Finally, the comparator landscape is shifting: higher-dose semaglutide formulations and next-generation multi-agonists will require re-evaluation of what is currently framed as a two-drug decision, and no included study reported cost-effectiveness or equity outcomes, although differential access and supply constraints plausibly exert a larger population-level influence than the pharmacological difference documented here.

This review has several strengths. The search was executed across four databases with supplementary backward and forward citation tracking and trial-registry screening; eligibility criteria were prespecified and applied strictly to original comparative research; screening, extraction, and appraisal were performed in duplicate; design-appropriate validated risk-of-bias instruments were applied separately to randomised and non-randomised evidence; and the decision not to pool was made transparently against prespecified criteria rather than by default. The corresponding limitations of the review and of the underlying evidence are set out in the following section.

Limitations

Several limitations apply to the conduct of this review. It was not prospectively registered in PROSPERO or another registry, so although an internal protocol was agreed in advance, readers cannot independently verify that the reported methods and outcomes match those planned. An English-language restriction was applied, which introduces potential language bias and may have excluded eligible non-English reports, plausibly from regions already under-represented in this evidence base. Grey literature was not searched, and conference abstracts and preprints were excluded by design; while this ensures that every included study has undergone peer review, it means the synthesis may lag the most recent evidence and cannot exclude publication bias, which in any case cannot be assessed statistically in the absence of a pooled analysis. Inter-rater agreement at the screening stage was not quantified using a chance-corrected statistic, so the reproducibility of study selection is not formally demonstrated. Further limitations are inseparable from the underlying evidence. Only one included study was randomised, and it was open-label, of 72 weeks' duration, and restricted to adults with obesity but without type 2 diabetes; all remaining evidence is retrospective and observational, and four of the 10 cohorts were judged at serious risk of bias, predominantly from unadjusted confounding. Even among the six cohorts judged at low risk, confounding by indication remains intrinsic to non-randomised evidence in this field, since prescribers select between these agents on the basis of anticipated response, comorbidity, formulary status, and supply. Requirements for treatment persistence and for available follow-up weight measurements introduce selection bias that plausibly favours patients responding well to therapy, and achieved dose was frequently inferred from dispensing records rather than measured. Comparative safety reporting was available in only four studies and is likely to be substantially incomplete in database-derived cohorts. Because the prespecified conditions for pooling were not met, no summary effect estimate and no statistical measure of heterogeneity or small-study effects is presented. Possible participant overlap between two cohorts sharing a common data platform, industry sponsorship of several included studies, and the concentration of evidence in North American populations further constrain generalisability. These findings should therefore be regarded as establishing a consistent and clinically relevant direction of effect rather than a precise quantification of comparative benefit.

## Conclusions

Tirzepatide consistently produced greater weight reduction than semaglutide, with the advantage most pronounced at stringent weight-reduction thresholds and accompanied by modestly greater improvements in blood pressure and glycated haemoglobin. The magnitude of benefit was substantially larger under randomised trial conditions than in routine care, where suboptimal dose escalation and frequent early discontinuation attenuated the outcomes achieved with both agents. Short-term safety appeared broadly comparable, with gastrointestinal events predominating, although comparative adverse event data were reported in only a minority of studies. Randomised evidence beyond 72 weeks, in populations with type 2 diabetes, and for hard cardiovascular endpoints remains absent. Tirzepatide can reasonably be regarded as the more effective of the two agents for weight reduction on current evidence, but treatment selection should also weigh the established cardiovascular outcome data for semaglutide, tolerability, cost, access, and the patient's capacity to sustain long-term therapy at an adequate dose, since persistence and dose attainment influenced outcomes at least as much as the choice between agents.

## References

[1] NCD Risk Factor Collaboration (NCD-RisC) (2024). Worldwide trends in underweight and obesity from 1990 to 2022: a pooled analysis of 3663 population-representative studies with 222 million children, adolescents, and adults. Lancet.

[2] Okunogbe A, Nugent R, Spencer G, Powis J, Ralston J, Wilding J (2022). Economic impacts of overweight and obesity: current and future estimates for 161 countries. BMJ Glob Health.

[3] Elmaleh-Sachs A, Schwartz JL, Bramante CT, Nicklas JM, Gudzune KA, Jay M (2023). Obesity management in adults: a review. JAMA.

[4] Wilding JP, Batterham RL, Calanna S (2021). Once-weekly semaglutide in adults with overweight or obesity. N Engl J Med.

[5] Jastreboff AM, Aronne LJ, Ahmad NN (2022). Tirzepatide once weekly for the treatment of obesity. N Engl J Med.

[6] Davies M, Faerch L, Jeppesen OK (2021). Semaglutide 2·4 mg once a week in adults with overweight or obesity, and type 2 diabetes (STEP 2): a randomised, double-blind, double-dummy, placebo-controlled, phase 3 trial. Lancet.

[7] Garvey WT, Frias JP, Jastreboff AM (2023). Tirzepatide once weekly for the treatment of obesity in people with type 2 diabetes (SURMOUNT-2): a double-blind, randomised, multicentre, placebo-controlled, phase 3 trial. Lancet.

[8] Lincoff AM, Brown-Frandsen K, Colhoun HM (2023). Semaglutide and cardiovascular outcomes in obesity without diabetes. N Engl J Med.

[9] Aronne LJ, Sattar N, Horn DB (2024). Continued treatment with tirzepatide for maintenance of weight reduction in adults with obesity: the SURMOUNT-4 randomized clinical trial. JAMA.

[10] Frías JP, Davies MJ, Rosenstock J (2021). Tirzepatide versus semaglutide once weekly in patients with type 2 diabetes. N Engl J Med.

[11] Page MJ, McKenzie JE, Bossuyt PM (2021). The PRISMA 2020 statement: an updated guideline for reporting systematic reviews. BMJ.

[12] Sterne JA, Savović J, Page MJ (2019). RoB 2: a revised tool for assessing risk of bias in randomised trials. BMJ.

[13] Sterne JA, Hernán MA, Reeves BC (2016). ROBINS-I: a tool for assessing risk of bias in non-randomised studies of interventions. BMJ.

[14] Rodriguez PJ, Goodwin Cartwright BM, Gratzl S, Brar R, Baker C, Gluckman TJ, Stucky NL (2024). Semaglutide vs tirzepatide for weight loss in adults with overweight or obesity. JAMA Intern Med.

[15] Jamal M, Alhashemi M, Dsouza C, Al-Hassani S, Qasem W, Almazeedi S, Al-Sabah S (2024). Semaglutide and tirzepatide for the management of weight recurrence after sleeve gastrectomy: a retrospective cohort study. Obes Surg.

[16] Anson M, Henney AE, Broadwell N (2024). Incidence of new onset type 2 diabetes in adults living with obesity treated with tirzepatide or semaglutide: real world evidence from an international retrospective cohort study. EClinicalMedicine.

[17] Trinh H, Donovan A, McAdam-Marx C (2025). Real-world effectiveness of tirzepatide versus semaglutide for weight loss in overweight or obese patients in an ambulatory care setting. Diabetes Obes Metab.

[18] Aronne LJ, Horn DB, le Roux CW (2025). Tirzepatide as compared with semaglutide for the treatment of obesity. N Engl J Med.

[19] Gasoyan H, Butsch WS, Schulte R (2025). Changes in weight and glycemic control following obesity treatment with semaglutide or tirzepatide by discontinuation status. Obesity (Silver Spring).

[20] Ng CD, Divino V, Wang J, Toliver JC, Buss M (2025). Real-world weight loss observed with semaglutide and tirzepatide in patients with overweight or obesity and without type 2 diabetes (SHAPE). Adv Ther.

[21] Shil KK, Hira AD, Bakchi S, Paul SK, Hossain M, Ghosh DK (2025). Efficacy and safety of tirzepatide and semaglutide for obesity management: a real-world comparison. Cureus.

[22] Al Ozairi E, Irshad M, Alkandari J, Sojan L, Alroudhan D, Alotaibi N, le Roux CW (2026). Weight loss in people with type 1 diabetes over 12 months: real-world data comparing tirzepatide, semaglutide and liraglutide. Diabetes Obes Metab.

[23] le Roux CW, Done N, Brnabic AJ (2026). Comparative effectiveness of tirzepatide and semaglutide for obesity management in US clinical practice: a 6-month retrospective cohort study. J Endocrinol Invest.

[24] Cetiner S (2026). Real-world effectiveness and safety of tirzepatide, semaglutide, and liraglutide in adults with overweight or obesity without diabetes: a comparative study. Diabetes Metab Syndr Obes.

[25] Munawar N, Mahato A, Rawat A (2025). Tirzepatide versus semaglutide for weight loss in overweight and obese adults: a systematic review and meta-analysis of direct comparative studies. Cureus.

[26] Krüger N, Schneeweiss S, Desai RJ (2026). Cardiovascular outcomes of semaglutide and tirzepatide for patients with type 2 diabetes in clinical practice. Nat Med.

[27] Nicholls SJ, Pavo I, Bhatt DL (2025). Cardiovascular outcomes with tirzepatide versus dulaglutide in type 2 diabetes. N Engl J Med.

